# Common Genetic Polymorphisms within NFκB-Related Genes and the Risk of Developing Invasive Aspergillosis

**DOI:** 10.3389/fmicb.2016.01243

**Published:** 2016-08-12

**Authors:** Carmen B. Lupiañez, María T. Villaescusa, Agostinho Carvalho, Jan Springer, Michaela Lackner, José M. Sánchez-Maldonado, Luz M. Canet, Cristina Cunha, Juana Segura-Catena, Laura Alcazar-Fuoli, Carlos Solano, Luana Fianchi, Livio Pagano, Leonardo Potenza, José M. Aguado, Mario Luppi, Manuel Cuenca-Estrella, Cornelia Lass-Flörl, Hermann Einsele, Lourdes Vázquez, PCRAGA Study Group, Rafael Ríos-Tamayo, Jurgen Loeffler, Manuel Jurado, Juan Sainz

**Affiliations:** ^1^Genomic Oncology Area, GENYO, Center for Genomics and Oncological Research, Pfizer/University of Granada/Andalusian Regional Government, PTS GranadaGranada, Spain; ^2^Hematology Department, Virgen de las Nieves University HospitalGranada, Spain; ^3^Hematology Department, University Hospital of SalamancaSalamanca, Spain; ^4^Hematology Department, Jiménez Díaz FoundationMadrid, Spain; ^5^Life and Health Sciences Research Institute (ICVS), School of Health Sciences, University of MinhoBraga, Portugal; ^6^ICVS/3B's - PT Government Associate LaboratoryBraga, Portugal; ^7^Universitätsklinikum Würzburg, Medizinische Klinik IIWürzburg, Germany; ^8^Division of Hygiene and Medical Microbiology, Medical University of InnsbruckInnsbruck, Austria; ^9^Mycology Reference Laboratory, Centro Nacional de Microbiología, Instituto de Salud Carlos IIIMadrid, Spain; ^10^Hematology Department, Clinic University Hospital of ValenciaValencia, Spain; ^11^Istituto di Ematologia, Università Cattolica del S. CuoreRome, Italy; ^12^Department of Medical and Surgical Sciences, University of Modena and Reggio EmiliaAOU Policlinico, Modena, Italy; ^13^Unit of Infectious Diseases, University Hospital 12 de Octubre, Research Institute of Hospital 12 de Octubre (i+12)Madrid, Spain; ^14^PCRAGA Study GroupSalamanca, Spain

**Keywords:** Invasive Aspergillosis, genetic polymorphisms, susceptibility, NFκB-related genes, interaction

## Abstract

Invasive Aspergillosis (IA) is an opportunistic infection caused by *Aspergillus*, a ubiquitously present airborne pathogenic mold. A growing number of studies suggest a major host genetic component in disease susceptibility. Here, we evaluated whether 14 single-nucleotide polymorphisms within *NF*κ*B1, NF*κ*B2, RelA, RelB, Rel*, and *IRF4* genes influence the risk of IA in a population of 834 high-risk patients (157 IA and 677 non-IA) recruited through a collaborative effort involving the aspBIOmics consortium and four European clinical institutions. No significant overall associations between selected SNPs and the risk of IA were found in this large cohort. Although a hematopoietic stem cell transplantation (HSCT)-stratified analysis revealed that carriers of the *IRF4*_rs12203592T/T_ genotype had a six-fold increased risk of developing the infection when compared with those carrying the C allele (OR_REC_ = 6.24, 95%CI 1.25–31.2, *P* = 0.026), the association of this variant with IA risk did not reach significance at experiment-wide significant threshold. In addition, we found an association of the IRF4_AATC_ and IRF4_GGTC_ haplotypes (not including the *IRF4*_rs12203592T_ risk allele) with a decreased risk of IA but the magnitude of the association was similar to the one observed in the single-SNP analysis, which indicated that the haplotypic effect on IA risk was likely due to the *IRF4*_rs12203592_ SNP. Finally, no evidence of significant interactions among the genetic markers tested and the risk of IA was found. These results suggest that the SNPs on the studied genes do not have a clinically relevant impact on the risk of developing IA.

## Introduction

Invasive Aspergillosis (IA) is an opportunistic infection often caused by species of *Aspergillus*, a common saprophytic filamentous fungus that is ubiquitously present in the environment. The LIFE initiative has estimated that around 30 million patients worldwide are at high risk of developing IA every year and over 200,000 patients develop the infection annually. Despite the substantial improvement in efficacy of newly developed anti-fungal drugs (mainly azoles alone or in combination with other antifungal drugs), IA continues to be a life-threatening infection in an increasing proportion of immunocompromised or critically ill subjects (Neofytos et al., 2009; Steinbach et al., 2012). IA is frequently encountered in patients of intensive care (Meersseman et al., 2007) and solid organ transplantation units (Singh et al., 2013) but also among those patients who undergo allogeneic hematopoietic stem cell transplantation (allo-HSCT) or are diagnosed either with acute myeloid leukemia (AML) or acute lymphoid leukemia (ALL) and receive high-dose chemotherapy regimens (Steinbach et al., 2012; Neofytos et al., 2013a,b).

Although major clinical risk factors for IA have been identified (Kousha et al., 2011) and the management of high-risk patients has been improved through the optimization of prevention strategies and early initiation of anti-fungal prophylaxis, mortality rates for IA remain still today unacceptably high (up to 30–60%; Neofytos et al., 2009; Steinbach et al., 2012; Karthaus and Buchheidt, 2013). These observational findings suggest that additional factors may contribute to the risk of developing IA. In this regard, a growing number of studies have suggested that host genetic polymorphisms within or near immune-related genes may contribute to determine the risk of developing the infection (Kesh et al., 2005; Sainz et al., 2007a,b, 2008a,b, 2010, 2012; Bochud et al., 2008; Mezger et al., 2008; Ramaprakash et al., 2009; Cunha et al., 2010, 2011, 2014; Chai et al., 2011; Grube et al., 2013; Stappers et al., 2014; Wojtowicz et al., 2015a,b). Interestingly, a substantial proportion of the susceptibility markers identified to date for IA are located in genes directly or indirectly implicated in the activation of the nuclear factor-kappa B (NFκB) signaling pathway, which suggests a relevant role of this biological route in determining the risk of developing this fungal infection. In particular, it has been reported that single nucleotide polymorphisms (SNPs) within toll-like receptors (TLRs; Mambula et al., 2002; Kesh et al., 2005; Bochud et al., 2008; Pamer, 2008; Ramaprakash et al., 2009; Carvalho et al., 2012; Grube et al., 2013), C-type lectins (Cunha et al., 2010; Chai et al., 2011; Sainz et al., 2012), PTX3 (Cunha et al., 2014), and tumor necrosis factor receptors (TNFRs; Sainz et al., 2007b, 2010), which are pathogen recognition receptors (PRRs) that often culminate in the activation of NFκB pathway, may render patients more susceptible to develop IA.

Based on these findings but also those that have demonstrated that *NF*κ*B1* (p105/p50), *NF*κ*B2* (p100/p52), Rel A (p65), RelB, c-Rel genes may form homo- and hetero-dimers to regulate the activation of the canonical and non-canonical NFκB pathways (Moynagh, 2005; Gilmore, 2006; Hoffmann et al., 2006; Schlitzer et al., 2013; Bajaña et al., 2016) but also IRF4-dependent immune responses (Boddicker et al., 2015), we hypothesized that the presence of common genetic polymorphisms within *NF*κ*B1, NF*κ*B2, RelA, RelB, Rel*, and *IRF4* genes might influence the risk of developing IA in high-risk patients. Thus, the aim of the present study was to investigate the relationship between 14 single nucleotide polymorphisms within these genes and the risk of IA but also to determine whether these variants could interact with each other to modify the risk of developing the infection.

## Material and methods

### Study population

Eight hundred and thirty-four high-risk European Caucasian patients undergoing allo-HSCT or being diagnosed with acute myeloid leukemia (AML) or acute lymphoid leukemia (ALL) and receiving intensive remission-induction chemotherapy were recruited in this case-control population-based study (Lupiañez et al., 2015). Three hundred and thirty-five patients were ascertained from the aspBIOmics consortium (http://www.aspbiomics.eu) whereas 341 patients were collected from two Spanish medical institutions (University Hospital of Salamanca and Clinic University Hospital of Valencia) and through a Spanish multicenter clinical trial (PCRAGA, EU clinical trial number: 2010-019406-17; Aguado et al., 2015). In addition, 148 patients were recruited from two Italian medical institutions (Università Cattolica del S. Cuore, Rome; and University of Modena and Reggio Emilia, AOU Policlinico, Modena). Of those 834 patients, a total of 157 patients were diagnosed with proven or probable IA according to the updated EORTC/MSG criteria (De Pauw et al., 2008) whereas the remaining 677 patients did not show any sign of fungal infection.

### SNP selection and genotyping

Fourteen polymorphisms within the *NF*κ*B1, NF*κ*B2, RelA, RelB, Rel*, and *IRF4* genes were selected to be genotyped in the whole population (Table 1). SNP selection was based on previously reported associations with cancer (Curtin et al., 2010; Do et al., 2010; Han et al., 2011; Slattery et al., 2011; Seufert et al., 2013; Wang et al., 2014) and immune-related diseases including infections (Chen et al., 2006, 2016; Gregersen et al., 2009; Trynka et al., 2009; Eyre et al., 2010; Varadé et al., 2011; Bowes et al., 2012; Ellinghaus et al., 2012; Ali et al., 2013; Leung et al., 2014; Pan et al., 2015) but also because their potential functionality according to the Haploreg (http://www.broadinstitute.org/mammals/haploreg/haploreg.php) and ENCODE annotation data (https://genome.ucsc.edu/ENCODE/). The genotyping of the selected polymorphisms was carried out at GENYO (Center for Genomics and Oncological Research: Pfizer/University of Granada/Andalusian Regional Government, Granada, Spain) using KASPar® assays (LGC Genomics, Hoddesdon, UK) according to manufacturer's instructions. For internal quality control, 5% of samples were randomly selected and included as duplicates. Concordance between the original and the duplicate samples for the 14 SNPs was ≥99.0%. Call rates for all SNPs were ≥90.0% with the exception of the *IRF4*_rs872071_ SNP that was excluded from further analysis.

**Table 1:** **NFκB-related polymorphisms**.

**Gene**	**dbSNP rs#**	**Chr**.	**Location/Aa change**	**Nucleotide substitution**	**Effect-allele**
NFκB1	rs4648110	4	Intronic	A/T	A
NFκB2	rs12769316	10	Near gene	A/G	A
	rs1056890	10	Near gene	C/T	T
	rs11574851	10	N698N	C/T	T
REL	rs13031237	2	Intronic	G/T	T
	rs842647	2	Intronic	A/G	G
	rs13017599	2	Near gene	A/G	A
RELA	rs7119750	11	Intronic	C/T	T
RELB	rs2288918	19	Intronic	C/T	C
IRF4	rs872071	6	3′-UTR	A/G	G
	rs1877175	6	3′-UTR	A/G	A
	rs1050975	6	3′-UTR	A/G	G
	rs7768807	6	3′-UTR	C/T	C
	rs12203592	6	Intronic	C/T	T

*SNP, Single Nucleotide Polymorphism; Aa, Aminoacid*.

### Statistical analysis

The Hardy-Weinberg Equilibrium (HWE) test was performed in the control group (non-IA patients) by a standard observed-expected chi-square (χ^2^). Logistic regression analysis adjusted for age, sex, country of origin, allo-HSCT, underlying disease and prophylaxis status was used to assess the main effects of the selected SNPs on IA risk. We also performed gene-HSCT interaction analyses to determine whether the association between SNPs and IA was of similar magnitude in HSCT (at highest risk) and non-HSCT patients. Although the selection of variables for adjustment was based on well-established risk factors for IA, the partial availability of data regarding prophylaxis status, type of chemotherapy, immunosuppressive drugs (HSCT), HLA mismatch (HSCT), or CMV status did not allow us to assess the impact of these factors on the genetic associations tested. Statistical power of the overall and HSCT-stratified analyses was estimated using Quanto software (http://hydra.usc.edu/gxe/). All tests were conducted using the statistical software SPSS (v.20) and STATA (v.12) for MAC.

In order to account for multiple comparisons, we calculated an adjusted significance level using the Meff method (Nyholt, 2004) but also considering the number of inheritance models tested (codominant, dominant, recessive, and log-additive). Thus, the significant threshold used for the main effect analysis was 0.001 ([0.05]/13 independent genetic markers]/4 inheritance models).

### Linkage disequilibrium (LD) and haplotype analysis

Haplotype blocks were constructed from the genotyping data of the non-IA control group using the SNPtool (http://www.dkfz.de/de/molgen_epidemiology/tools/SNPtool.html; Chen et al., 2009) and the Haploview software (v.4.2). Selected polymorphisms within the same locus were not in linkage disequilibrium ensuring that these variants represented independent variability signals (Supplementary Figure 1). In addition to the analysis based on a single SNP, we performed haplotype frequency estimation and haplotype association analysis using SNPStats (http://bioinfo.iconcologia.net/SNPstats). Haplotype frequencies were determined using the Expectation-maximization (EM) algorithm and the minimum haplotype frequency was set at 0.01, therefore assessing association for common haplotypes.

### eQTL analysis

We also assessed whether selected polymorphisms correlated with mRNA expression in the publicly available eQTL IGV browser for primary cells (http://www.gtexportal.org/home/) or using the Haploreg data.

### SNP-SNP interaction analysis

We also were interested in testing whether NFκB-related SNPs could interact with each other to modify the risk of IA using the multifactor dimensionality reduction (MDR) software. A detailed description of the MDR method has been reported elsewhere (Ritchie et al., 2003; Moore, 2004). A 10-fold cross-validation analysis (exhaustive search) and permutation testing were used to confirm the best interaction models. MDR models were considered statistically significant at *P* < 0.05 (*P* sign). Statistical significance of each particular model was then re-evaluated using a 1.000-fold permutation test to compare observed testing balanced accuracies with those expected under the null hypothesis of no association (using the MDR permutation testing module 0.4.9 alpha). Interactions were visualized by constructing an interaction dendrogram according to the method described by Moore et al. (Moore, 2004). MDR software and MDR permutation testing module are open-source and freely available from http://www.epistasis.org.

## Results

In this population-based case-control study a total of 834 hematological patients were recruited. Demographic and clinical characteristics of these patients are summarized in Table 2. IA and non-IA patients had similar age but male patients were more prone to develop IA than females (male/female ratio = 1.86 vs. 1.18, *P* = 0.015). HSCT was common and equally distributed between IA and non-IA groups (45.85 vs. 45.49%) and the underlying disease (hematological disorder) was also uniform between both groups (Table 2). As expected, patients without prophylaxis were more prone to develop IA compared to those patients who take antifungal drugs (23.28 vs. 14.51%; Table 2).

**Table 2:** **Demographic and clinical characteristic of IA and non-IA patients**.

**aspBIOmics consortium + UHS-GHV-PCR-AGA-UCSC-MORE populations**				
	**Overall (*n* = 834)**	**IA patients (*n* = 157)**	**Non-IA patients (*n* = 677)**	***P*-value**
**DEMOGRAPHIC VARIABLES**				
Age	52.89 ± 15.95	53.78 ± 15.37	52.69 ± 16.07	NS
Sex ratio (male/female)	1.28 (466/362)	1.86 (99/53)	1.18 (367/309)	**0.015**
**HEMATOLOGICAL DISEASE**				
AML	571 (68.62)	112 (71.33)	459 (68.00)	NS
ALL	77 (09.25)	18 (11.46)	59 (08.74)	NS
Other	184 (22.11)	27 (17.19)	157 (23.25)	NS
HSCT	380 (45.56)	72 (45.85)	308 (45.49)	NS
PROPHYLAXIS^*^				
Ever use of prophylaxis	386 (46.28)	56 (35.67)	330 (48.74)	**0.006**
Never use of prophylaxis	232 (27.81)	54 (34.39)	178 (26.29)	**0.006**

*HSCT, Hematopoietic stem cell transplantationl; AML, acute myeloid leukemia; ALL, acute lymphoid leukemia; UHS, University hospital of Salamanca (Spain); GHV, General hospital of Valencia (Spain); PCRAGA clinical trial (EU clinical trial number: 2010-019406-17); UCSC, Università Cattolica del S. Cuore, Rome; MORE, University of Modena and Reggio Emilia, AOU Policlinico, Modena (Italy). P values ≤ 0.05 were considered significant and are highlighted in bold*.

* *Some patients had several prophylactic drugs*.

*Data on underlying disease and sex was not available for 2 and 6 patients, respectively*.

*Prophylaxis status was unknown for 216 patients*.

All SNPs were in Hardy-Weinberg in the control group with the exception of the *REL*_rs13031237_ that was excluded from further analyses (non-IA; *P* < 0.001). Allele and genotype frequencies of selected SNPs were in line with those reported in Hapmap. Logistic regression analysis adjusted for age, sex, country of origin, allo-HSCT, and underlying disease showed that none of the selected SNPs was significantly associated with the risk of IA according to codominant, dominant, recessive and log-additive models of inheritance (Table 3). In addition, although prophylaxis status was only partially available in our population (*n* = 618), no significant changes in association estimates were observed when this clinical variable was included as covariate for adjustment (data not shown). No significant associations between the *NF*κ*B1, NF*κ*B2, cRel, RelB*, and *IRF4* polymorphisms and IA risk were also found in an allo-HSCT-restricted analysis considering donor genotypes and IA episodes occurred after transplantation (Table 4). Although our statistical power was limited (Supplementary Table 1), we found a fairly weak association of the *IRF4*_rs12203592T/T_ genotype with the risk of developing the infection at the nominal significance level of *P* < 0.05. Carriers of the *IRF4*_rs12203592T/T_ genotype showed a six-fold increased risk of developing IA when compared with patients carrying the wild type C-allele (OR_REC_ = 6.24, 95%CI 1.25–31.2, *P* = 0.026). Interestingly, we found that the presence of the minor allele of the rs12203592 (T) strongly correlated with *IRF4* mRNA expression levels in whole blood samples and Epstein-Barr virus (EBV)-transformed lymphocytes (*P* = 6.0 • 10^−7^ and *P* = 3.0 • 10^−7^, respectively), which suggested a possible functional role of this polymorphism. Based on these interesting results, we decided to explore the potential functional impact of this polymorphism using Haploreg and ENCODE annotation data. This analysis revealed that the *IRF4*_rs12203592_ SNP resides on a strong enhancer and near of an active promoter that might constitute a regulatory element for IRF4. In addition, this analysis showed that this intronic polymorphism was predicted to change binding motifs for NFκB, HDAC2, and HMG-IY, which are proteins implicated in the transcriptional regulation of multiple innate and adaptive immune-related genes (Liu et al., 2001; Hayden et al., 2006; Falkenberg and Johnstone, 2014). In line with the findings suggesting a functional role of this variant, we also found that the rs12203592 SNP mapped among promoter and enhancer histone marks in different primary T-cell subtypes (TCD8+, TCD4+, Treg, and Th17) of different origin (peripheral blood, hematopoietic stem cells, cord blood, etc.) but also in primary B-cells, natural killer cells and monocytes. Although at this point it was tempting to speculate that the *IRF4*_rs12203592_ SNP might play a role in modulating the risk of developing IA likely through the regulation of *IRF4* mRNA expression levels, the association of this SNP with IA risk did not remained significant after correction for multiple testing (*P* = 0.001; Table 4) and, therefore, requires further confirmation. In agreement with the single-SNP analysis, haplotype association analysis showed fairly weak associations of the IRF4_AATC_ and IRF4_GGTC_ haplotypes (not including the *IRF4*_rs12203592T_ risk allele) with a decreased risk of IA. However, these associations were of similar magnitude to the one observed for the *IRF4*_rs12203592_ SNP in the single-SNP analysis (OR = 0.28, 95%CI 0.08–0.95, *P* = 0.042 and OR = 0.04, 95%CI 0.00–0.71; Table 5) suggesting that the haplotypic effect was due to this intronic variant.

**Table 3:** **Association estimates for NFκB-related polymorphisms and IA**.

**Variant_dbSNP**	**Gene**	**Effect-allele**	**OR (95% CI)^a^**	***P_value_***	**OR (95% CI)^b^**	***P_value_***	**OR (95% CI)^c^**	***P_value_***
rs4648110	NFκB1	A	0.69 (0.46–1.03)	0.07	0.73 (0.30–1.81)	0.50	0.74 (0.53–1.04)	0.08
rs12769316	NFκB2	A	0.87 (0.57–1.31)	0.50	0.79 (0.23–2.73)	0.70	0.87 (0.61–1.26)	0.48
rs1056890	NFκB2	T	1.41 (0.94–2.10)	0.10	1.01 (0.58–1.76)	0.96	1.19 (0.90–1.57)	0.23
rs11574851	NFκB2	T	1.52 (0.89–2.60)	0.13	1.47 (0.15–14.5)	0.74	1.46 (0.89–2.40)	0.14
rs842647	cREL	G	0.92 (0.63–1.34)	0.67	1.03 (0.50–2.14)	0.93	0.95 (0.71–1.29)	0.77
rs13017599	cREL	A	0.78 (0.54–1.13)	0.18	1.12 (0.67–1.88)	0.66	0.90 (0.69–1.18)	0.47
rs7119750	RELA	T	0.94 (0.61–1.46)	0.80	0.90 (0.19–4.26)	0.89	0.95 (0.64–1.41)	0.79
rs2288918	RELB	C	1.18 (0.80–1.73)	0.40	0.57 (0.30–1.09)	0.09	0.97 (0.73–1.28)	0.81
rs1877175	IRF4	A	0.82 (0.56–1.22)	0.34	0.59 (0.22–1.58)	0.30	0.82 (0.59–1.14)	0.24
rs1050975	IRF4	G	1.00 (0.61–1.63)	0.99	2.69 (0.75–9.61)	0.13	1.10 (0.72–1.68)	0.67
rs7768807	IRF4	C	1.33 (0.92–1.94)	0.13	1.26 (0.66–2.40)	0.48	1.24 (0.93–1.65)	0.14
rs12203592	IRF4	T	1.01 (0.66–1.53)	0.97	2.14 (0.64–7.19)	0.22	1.07 (0.74–1.56)	0.72

*OR, odds ratio; CI, confidence interval; SNP, single nucleotide polymorphism. Estimates were adjusted for age, sex, country of origin, allo-SCT, and underlying disease. P < 0.0011 was defined as multiple testing significance threshold*.

a *Estimates according to a dominant model of inheritance*.

b *Estimates according to a recessive model of inheritance*.

c *Estimates according to an additive model of inheritance*.

**Table 4:** **Association estimates for NFκB-related polymorphisms and IA in HSCT patients (***n*** = 239)**.

**Variant_dbSNP**	**Gene**	**Effect-allele**	**OR (95% CI)^a^**	***P_value_***	**OR (95% CI)^b^**	***P_value_***	**OR (95% CI)^c^**	***P_value_***
rs4648110	NFκB1	A	1.04 (0.51–2.12)	0.92	2.99 (0.65–13.7)	0.16	1.19 (0.65–2.18)	0.57
rs12769316	NFκB2	A	1.29 (0.60–2.76)	0.51	NA (NA–NA)	NA	1.16 (0.57–2.37)	0.67
rs1056890	NFκB2	T	1.42 (0.67–3.00)	0.36	1.32 (0.44–3.96)	0.63	1.29 (0.75–2.24)	0.36
rs11574851	NFκB2	T	1.59 (0.56–4.53)^*^	0.38	NA (NA–NA)	NA	NA (NA–NA)	NA
rs842647	cREL	G	0.81 (0.40–1.64)	0.55	0.94 (0.23–3.74)	0.93	0.86 (0.48–1.53)	0.61
rs13017599	cREL	A	0.84 (0.42–1.68)	0.62	1.50 (0.61–3.67)	0.38	1.03 (0.63–1.68)	0.91
rs7119750	RELA	T	0.53 (0.22–1.24)	0.14	NA (NA–NA)	NA	0.52 (0.23–1.18)	0.12
rs2288918	RELB	C	1.17 (0.58–2.36)	0.66	0.86 (0.33–2.26)	0.76	1.04 (0.64–1.68)	0.88
rs1877175	IRF4	A	0.68 (0.32–1.44)	0.32	0.69 (0.08–6.00)	0.74	0.72 (0.37–1.40)	0.33
rs1050975	IRF4	G	0.65 (0.26–1.66)	0.37	2.60 (0.37–18.4)	0.34	0.84 (0.39–1.80)	0.66
rs7768807	IRF4	C	0.73 (0.35–1.51)	0.39	0.95 (0.30–3.03)	0.93	0.82 (0.47–1.44)	0.49
rs12203592	IRF4	T	1.04 (0.49–2.20)	0.91	**6.24 (1.25–31.2)**	**0.026**	1.33 (0.72–2.47)	0.36

*OR, odds ratio; CI, confidence interval; SNP, single nucleotide polymorphism; NA, not applicable. Estimates were adjusted for age, sex, country of origin, and underlying disease. P < 0.05 in bold. P < 0.0011 was defined as multiple testing significance threshold*.

a *Estimates calculated according to a dominant model of inheritance*.

b *Estimates calculated according to a recessive model of inheritance*.

c *Estimates calculated according to an additive model of inheritance*.

* *Association estimates are referred to heterozygotes. Homozygotes for the rare allele were not found in the HSCT cohort*.

**Table 5:** **Haplotype association analysis and risk of IA**.

**Overall population (*N* = 834)**							**HSCT population (*N* = 239)**						
*NFκB2*rs1056890	*NFκB2*rs11574851	*NFκB2*rs12769316		**Freq**	**OR(95%CI)**^a^	***P*-value**	*NFκB2*rs1056890	*NFκB2*rs11574851	*NFκB2*rs12769316		**Freq**	**OR(95%CI)**^b^	***P*-value**
C	C	G		0.4708	1.00	–	C	C	G		0.4827	1.00	–
T	C	G		0.3507	1.22 (0.91–1.64)	0.19	T	C	G		0.3443	1.58 (0.87–2.85)	0.13
C	C	A		0.1115	0.74 (0.45–1.23)	0.25	C	C	A		0.1106	1.45 (0.58–3.62)	0.43
C	T	A		0.0517	1.70 (0.99–2.90)	0.053	C	T	A		0.0458	2.26 (0.73–7.02)	0.16
*REL*rs842647	*REL*rs13017599			**Freq**	**OR(95%CI)**^a^	***P*-value**	*REL*rs842647	*REL*rs13017599			**Freq**	**OR(95%CI)**^b^	***P*-value**
A	G			0.3786	1.00	–	A	A			0.3685	1.00	–
A	A			0.3640	0.76 (0.55–1.05)	0.10	A	G			0.3526	1.46 (0.78–2.74)	0.24
G	G			0.2473	0.76 (0.53–1.09)	0.14	G	G			0.2595	0.83 (0.41–1.68)	0.61
*IRF4rs1050975*	*IRF4rs1877175*	*IRF4rs7768807*	*IRF4rs12203592*	**Freq**	**OR(95%CI)**^a^	***P*-value**	*IRF4rs1050975*	*IRF4rs1877175*	*IRF4rs7768807*	*IRF4rs12203592*	**Freq**	**OR(95%CI)**^b^	***P*-value**
A	G	T	C	0.4034	1.00	–	A	G	T	C	0.3536	1.00	–
A	G	C	C	0.1948	1.32 (0.90–1.95)	0.16	A	G	C	C	0.2236	0.73 (0.34–1.56)	0.41
A	G	T	T	0.1094	1.12 (0.64–1.93)	0.70	A	A	T	C	0.1240	0.28 (0.08–0.95)	0.042
A	A	T	C	0.1075	0.76 (0.42–1.40)	0.39	A	G	T	T	0.1214	0.95 (0.38–2.37)	0.92
A	A	C	C	0.0718	0.89 (0.48–1.65)	0.71	G	G	T	C	0.0726	0.04 (0.00–0.71)	0.029
G	G	T	C	0.0716	0.74 (0.36-1.52)	0.41	A	A	C	C	0.0456	0.04 (0.00–111.9)	0.42

a *Estimates calculated according to a dominant model of inheritance and adjusted for age, sex, country of origin, allo-SCT and underlying disease*.

b *Estimates calculated according to a dominant model of inheritance and adjusted for age, sex, country of origin and underlying disease*.

**Figure 1:**
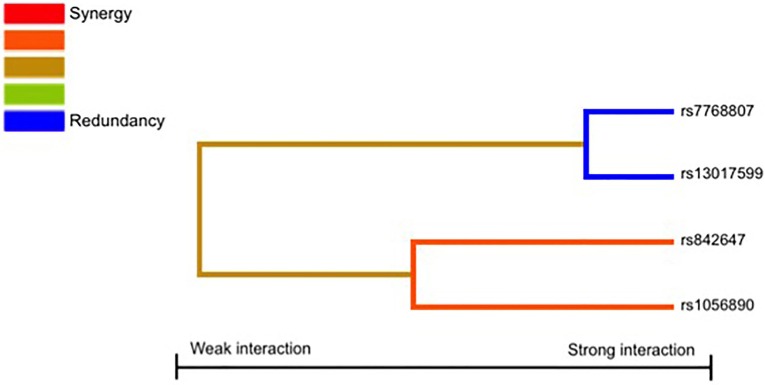
**Interaction dendrogram**. The interaction dendrogram reveals no significant interactions among the studied SNPs to modulate the risk of IA.

Finally, given the involvement of physiological complexes (p50/p65, p52/RelB, and p65-c-Rel) in the activation of the NFκB signaling pathway and the role of the p52/RelB complex in the transcriptional activation of the IRF4 (Boddicker et al., 2015), we also decided to investigate whether NFκB-related SNPs might interact to each other to modify the risk of IA. Results of the MDR analysis evaluating all possible combinations among the NFκB-related SNPs are shown in Table 6 and Figure 1. The best model suggested an interaction between the *REL*_rs842647_, *NF*κ*B2*_rs1056890_, *IRF4*_rs7768807_, and *REL*_rs13017599_ SNPs to synergistically increase the risk of developing IA (TA = 0.5488, P_Sign_ = 0.0010). Although this model was the best to predict IA and presented a high cross validation consistency (CVC) (9/10), it did not remain statistically significant following 1000-fold permutation test (*P* = 0.31). In addition, we found a significant 2-locus interaction model including the *NF*κ*B2*_rs1056890_ and *IRF4*_rs7768807_ SNPs to increase the risk of getting the infection (TA = 0.5353, P_Sign_ = 0.0010) but this interaction model also failed to retain statistical significance after 1000-fold permutation test (*P* = 0.47; Table 6). None of the best models included the *IRF4*_rs12203592_ SNP.

**Table 6:** **Multifactor dimensionality reduction analysis summary**.

	**Model**	**TA**	**Sign test (*P*-value)**	*P*-value^*^	**CVC**
1	*IRF4*_rs7768807_	0.5408	9 (0.0107)	0.39	9/10
2	*NF_κ_B2*_rs1056890_, *IRF4*_rs7768807_	0.5353	10 (0.0010)	0.47	8/10
3	*REL*_rs842647_, *NFκB2*_rs1056890_, *IRF4*_rs7768807_	0.4996	6 (0.0547)	0.84	4/10
4	*REL*_rs842647_, *NF_κ_B2*_rs1056890_, *IRF4*_rs7768807_, *REL*_rs13017599_	0.5488	10 (0.0010)	0.31	9/10

*TA, Testing accuracy; CVC, Cross-validation consistency*.

*Cross-validation and permutation testing were used to identify the best models. All possible two-way SNP interactions were tested using 10-fold cross-validation and the exhaustive search. The model with the highest testing balanced accuracy (TA) and cross validation consistency (CVC) was selected as “best model.” Statistical significance was evaluated by the Sign test and confirmed using a 1.000-fold permutation test to compare observed testing balanced accuracies with those expected under the null hypothesis of no association (using the MDR permutation testing module 0.4.9 alpha)*.

* *1000-fold permutation test (α = 0.001, TA = 0.6370; α = 0.01, TA = 0.5984; α = 0.05, TA = 0.5830; α = 0.10, TA = 0.5717)*.

## Discussion

The NFκB pathway is implicated in fostering a wide variety of physiological processes such as immune cell turnover (Hayden et al., 2006), inflammation (Karin and Greten, 2005), T-cell differentiation (Th1, Th2, Th17, and Treg subsets; Oh and Ghosh, 2013), DC maturation (Burkly et al., 1995; Abe et al., 2003), cell apoptosis (Wang et al., 1998; Karin and Greten, 2005), cell adhesion (Chen et al., 1995; Lockyer et al., 1998; Chiang et al., 2008), and angiogenesis (Beinke and Ley, 2004). In the presence of fungal pathogens such as *Aspergillus fumigatus*, the canonical NFκB pathway involving NEMO-IKKα-IKKβ and NFκB-p50/p65 complexes can be activated by membrane pattern recognition receptors (PRRs) such as toll-like receptors (TLRs; Mambula et al., 2002; Bellocchio et al., 2004; Roeder et al., 2004), C-type lectin receptors (CLRs; Serrano-Gómez et al., 2004; LeibundGut-Landmann et al., 2007; Gringhuis et al., 2009; Rivera et al., 2011; Saijo and Iwakura, 2011), cytokine receptors (IL1R, TNFRs) (Bozza et al., 2014), or soluble PRRs like pentraxins (PTX3; Garlanda et al., 2002) that leads to the immediate secretion of pro-inflammatory cytokines (TNF, IL1α, IL1β, IL6, IL12; Nicholson et al., 1996; Roeder et al., 2004), chemokines (CCL2, CCL3, CXCL2 and CXCL10; Shahan et al., 1998; Phadke and Mehrad, 2005; Reid et al., 2009), and reactive oxygen species (ROS; superoxide, hydroxyl radical, nitric oxide; Philippe et al., 2003; Chiang et al., 2008) but also to the expression of specific immune receptors and cell surface adhesion molecules (VCAM-1, E-Selectin; Akira et al., 2001; Chiang et al., 2008; Gringhuis et al., 2009). In addition, binding of fungal compounds to C-type lectins (Dectin-1 and Dectin-2) and the activation of certain TNF family cytokines (CD40L, BAFF, or LT-β) observed during fungal infections leads to the activation of the non-canonical NFκB pathway (p52/RelB), which is implicated in B-cell and DC maturation (Hu et al., 2011; Sun, 2012) and γδTh17 cell development (Powolny-Budnicka et al., 2011) but also in the control of the expression of a wide range of immune-related genes (Geijtenbeek and Gringhuis, 2009; Vallabhapurapu and Karin, 2009; Plato et al., 2013). On the other hand, it has recently been suggested the existence of a CD30-p52/Relb-IRF4 loop to regulate the NFκB pathway and cell proliferation (Boddicker et al., 2015) and a role of IRF4 in modulating the differentiation of different DC (Bajaña et al., 2016) and Th17-mediated immune responses against *A. fumigatus* (Schlitzer et al., 2013).

Considering the central role of the PRRs-NFκB pathways and IRF4 in the immune responses against *A. fumigatus* (Oh and Ghosh, 2013; Schlitzer et al., 2013; Williams et al., 2013) and those studies suggesting that genetic host factors may account for differences in susceptibility to IA (Ok et al., 2011; van der Velden et al., 2011), we decided to investigate the link between genetic polymorphisms within *NF*κ*B1, NF*κ*B2, RelA, RelB, Rel*, and *IRF4* genes and the risk of IA. In spite of the growing number of studies assessing the role of genetic polymorphisms within PRRs (*TLR2, TLR4, TLR9, Dectin-1, Dectin-2, DC-SIGN, PTX3*; Kesh et al., 2005; Bochud et al., 2008; Pamer, 2008; Cunha et al., 2010, 2011, 2014; Chai et al., 2011; Sainz et al., 2012; Grube et al., 2013; Wojtowicz et al., 2015b), cytokines (*IL1, IL10, IFNG*; Sainz et al., 2007a, 2008a; Lupiañez et al., 2015), and their receptors (*IL4R, TNFR1*, and *TNFR2*; Sainz et al., 2007b, 2010; Lupiañez et al., 2015) in determining the susceptibility to invasive fungal infections, this is the first study that attempts to evaluate the impact of polymorphisms within *NF*κ*B1, NF*κ*B2, RelA, RelB, REL*, and *IRF4* genes on the risk of developing IA. Our data showed no significant overall associations between selected SNPs and IA infection. The best result was the association of the *IRF4*_rs12203592_ SNP with an increased risk of IA in HSCT patients that suggested a weak effect of this locus on the risk of IA that might become evident only in those patients with a more profound degree of immunosuppression. However, despite the potential interest of these results and the evidences supporting a functional role of the *IRF4*_rs12203592_ variant in regulating *IRF4* mRNA expression and thereby modulating the NFκB pathway (Boddicker et al., 2015) and IRF4-dependent immune responses (Schlitzer et al., 2013; Bajaña et al., 2016), the association of this variant did not remain significant at the experiment-wide significance threshold, suggesting that the association of this polymorphism with IA could be due to chance. Haplotype analysis showed a small effect of common haplotypes on IA risk but the magnitude of these effects suggested that the observed association was likely due to the *IRF4*_rs12203592_ SNP rather than the combined effect of the *IRF4* SNPs. In addition, when we evaluated whether there was any significant interaction among the SNPs analyzed, we did not observe any consistent interaction model that could affect the risk of developing IA.

In conclusion, this case-control study does not provide strong evidence of a relationship between polymorphisms within *NF*κ*B1, NF*κ*B2, cRel, RelB*, and *IRF4* genes and IA risk. Nonetheless, given the limited statistical power of the HSCT-stratified analysis (80% to detect odds ratio of 2.1 at α = 0.001 for a SNP with a frequency of 0.25, dominant model) and the evidences suggesting a functional role of the *IRF4*_rs12203592_ SNP, we cannot dismiss the possibility of a small but still real effect of this variant or its haplotypes on the risk of IA in HSCT patients. Future case-control population-based studies conducted in larger HSCT populations are now warranted to further evaluate whether the *IRF4* locus may have a role in determining the susceptibility to IA.

## Ethics statement

The study protocol was approved by the local ethics review boards of all participating centers and written informed consent was obtained from each patient before inclusion in accordance with the Declaration of Helsinki. Ethical approval for this study was provided by the Comité de Ética e Investigación Clinica (CEIC) of the Virgen de las Nieves Hospital (Granada), University Hospital of Salamanca (Salamanca), Clinic University Hospital of Valencia (Valencia), and the Centro Nacional de Microbiologia (27_2012). The PCRAGA trial is registered with ClinicalTrials.gov (NCT01742026) and EudraCT (2010-019406-17). Ethical approval was also provided by the Subcomissao de Etica para as Ciencias da Vida e Saude (SECVS), University of Minho (approval SECVS 125/2014), (23533/16), the Comitato Etico Provinciale di Modena, the Ethics Committee of the Medical Faculty of the University of Wuerzburg (42/06) and the Ethic votum of the Medical University Innsbruck (UN4529).

## Author contributions

MJ and JS conceived the study and participated in its design and coordination. CBL performed the genetic analyses. TV, AC, JSp, ML, JMS-M, LMC, CC, JS-C, LA-F, CS, LF, LP, LPo, JMA, MLu, MC-E, CL-F, HE, LV, RR-T, JL, MJ and PCRAGA Study Group coordinated patient's recruitment and provided the clinical data. JS analysed the data. MJ and JS drafted the manuscript. All authors read and approved the final version of the manuscript.

## Funding

This study was supported by grants PI12/02688 from Fondo de Investigaciones Sanitarias (Instituto de Salud Carlos III, Madrid, Spain), the ERA-NET PathoGenoMics (03159000A; Ministerio de Ciencia e Innovación PIM2010EPA-00756, Madrid, Spain), the Collaborative Research Center / Transregio 124 FungiNet, the Austrian Science Fundation (FWF I-656-B09), the Fundação para a Ciência e Tecnologia (FCT), cofunded by Programa Operacional Regional do Norte (ON.2—O Novo Norte), the Quadro de Referência Estratégico Nacional (QREN) through the Fundo Europeu de Desenvolvimento Regional (FEDER) and the Projeto Estratégico – LA 26 – 2013–2014 (PEst-C/SAU/LA0026/2013). Agostinho Carvalho and Cristina Cunha were supported by the Fundação para a Ciência e Tecnologia (FCT), Portugal (IF/00735/2014 and SFRH/BPD/96176/2013, respectively). The PCRAGA trial was supported by an unrestricted grant from Pfizer, which had no involvement or control over the collection, analysis, and interpretation of data; the writing of the report; or the decision to submit the paper for publication. This study was also supported by Astellas Pharma Inc. and a donation from Consuelo González Moreno, an acute myeloid leukemia survivor.

### Conflict of interest statement

Dr. MC has been an advisor/consultant to the Panamerican Health Organization, Astellas Pharma, Gilead Sciences, Merck Sharp & Dohme, Pfizer, and Schering Plough. He has been paid for talks on behalf of Gilead Sciences, Merck Sharp & Dohme, Pfizer, Astellas Pharma, and Schering Plough. The other authors declare that the research was conducted in the absence of any commercial or financial relationships that could be construed as a potential conflict of interest.

## Supplementary material

Supplementary Table 1 **Statistical power calculations for NFκB-related polymorphisms**. SNP, Single Nucleotide Polymorphism; MAF, Aa, Aminoacid; ND, not determined; NP, no statistical power. Assumptions: IA incidence of 3.5% (Morgan et al. Med Mycol 2005; 43:S49-S58) and an α = 0.001 (study-wide significance). ^*^The statistical power was calculated based on genotype frequencies and assuming a dominant model of inheritance. ^*^The statistical power was calculated based on genotype frequencies and assuming a recessive model of inheritance.

Supplementary Figure 1 **Linkage disequilibrium (LD) blocks in the NFκB-related genes calculated in our population**. Numbers into squares indicate r^2^ values.
