# Supplementary material for: Common Genetic Polymorphisms within NFκB-Related Genes and the Risk of Developing Invasive Aspergillosis
Source: Front Microbiol. 2016 Aug 12;7:1243. doi: 10.3389/fmicb.2016.01243 (PMC4982195; doi:10.3389/fmicb.2016.01243)
Supplement: Supplementary Table 1 — Statistical power calculations for NFκB-related polymorphisms. SNP, Single Nucleotide Polymorphism; MAF, Aa, Aminoacid; ND, not determined; NP, no statistical power. Assumptions: IA incidence of 3.5% (Morgan et al. Med Mycol 2005; 43:S49-S58) and an α = 0.001 (study-wide significance). *The statistical power was calculated based on genotype frequencies and assuming a dominant model of inheritance. *The statistical power was calculated based on genotype frequencies and assuming a recessive model of inheritance. [file Table1.docx]

**Supplementary Table 1.** Statistical power calculations for *NFKB*-related polymorphisms.

| **Gene** | **dbSNP rs#** | **Chr.** | **Location/Aa change** | **Nucleotide substitution** | **Effect-allele** | **MAF** | **80% power to detect OR*** | **80% power to detect OR^‡^** |
| --- | --- | --- | --- | --- | --- | --- | --- | --- |
| *NFKB1* | rs4648110 | 4 | Intronic | A/T | A | 0.197 | 2.10 | 3.95 |
| *NFKB2* | rs12769316 | 10 | Near gene | A/G | A | 0.159 | 2.15 | 4.90 |
|  | rs1056890 | 10 | Near gene | C/T | T | 0.334 | 2.20 | 2.65 |
|  | rs11574851 | 10 | N698N | C/T | T | 0.053 | 2.70 | NP |
| *REL* | rs13031237 | 2 | Intronic | G/T | T | 0.349 | 2.20 | 2.55 |
|  | rs842647 | 2 | Intronic | A/G | G | 0.239 | 2.10 | 3.35 |
|  | rs13017599 | 2 | Near gene | A/G | A | 0.355 | 2.20 | 2.55 |
| *RELA* | rs7119750 | 11 | Intronic | C/T | T | 0.123 | 2.20 | 6.65 |
| *RELB* | rs2288918 | 19 | Intronic | C/T | C | 0.344 | 2.20 | 2.60 |
| *IRF4* | rs872071 | 6 | 3’-UTR | A/G | G | ND | ND | ND |
|  | rs1877175 | 6 | 3’-UTR | A/G | A | 0.200 | 2.10 | 3.90 |
|  | rs1050975 | 6 | 3’-UTR | A/G | G | 0.086 | 2.40 | 11.30 |
|  | rs7768807 | 6 | 3’-UTR | C/T | C | 0.257 | 2.10 | 3.15 |
|  | rs12203592 | 6 | Intronic | C/T | T | 0.135 | 2.20 | 6.00 |

Abbreviations: SNP, Single Nucleotide Polymorphism; MAF, Aa, Aminoacid; ND, not determined; NP, no statistical power.

Assumptions: IA incidence of 3.5% (Morgan *et al.* Med Mycol 2005; 43:S49-S58) and an α=0.001 (study-wide significance).

*The statistical power was calculated based on genotype frequencies and assuming a dominant model of inheritance.

*The statistical power was calculated based on genotype frequencies and assuming a recessive model of inheritance.
