# Supplementary figures and images for: Common Genetic Polymorphisms within NFκB-Related Genes and the Risk of Developing Invasive Aspergillosis
Source: Front Microbiol. 2016 Aug 12;7:1243. doi: 10.3389/fmicb.2016.01243 (PMC4982195; doi:10.3389/fmicb.2016.01243)

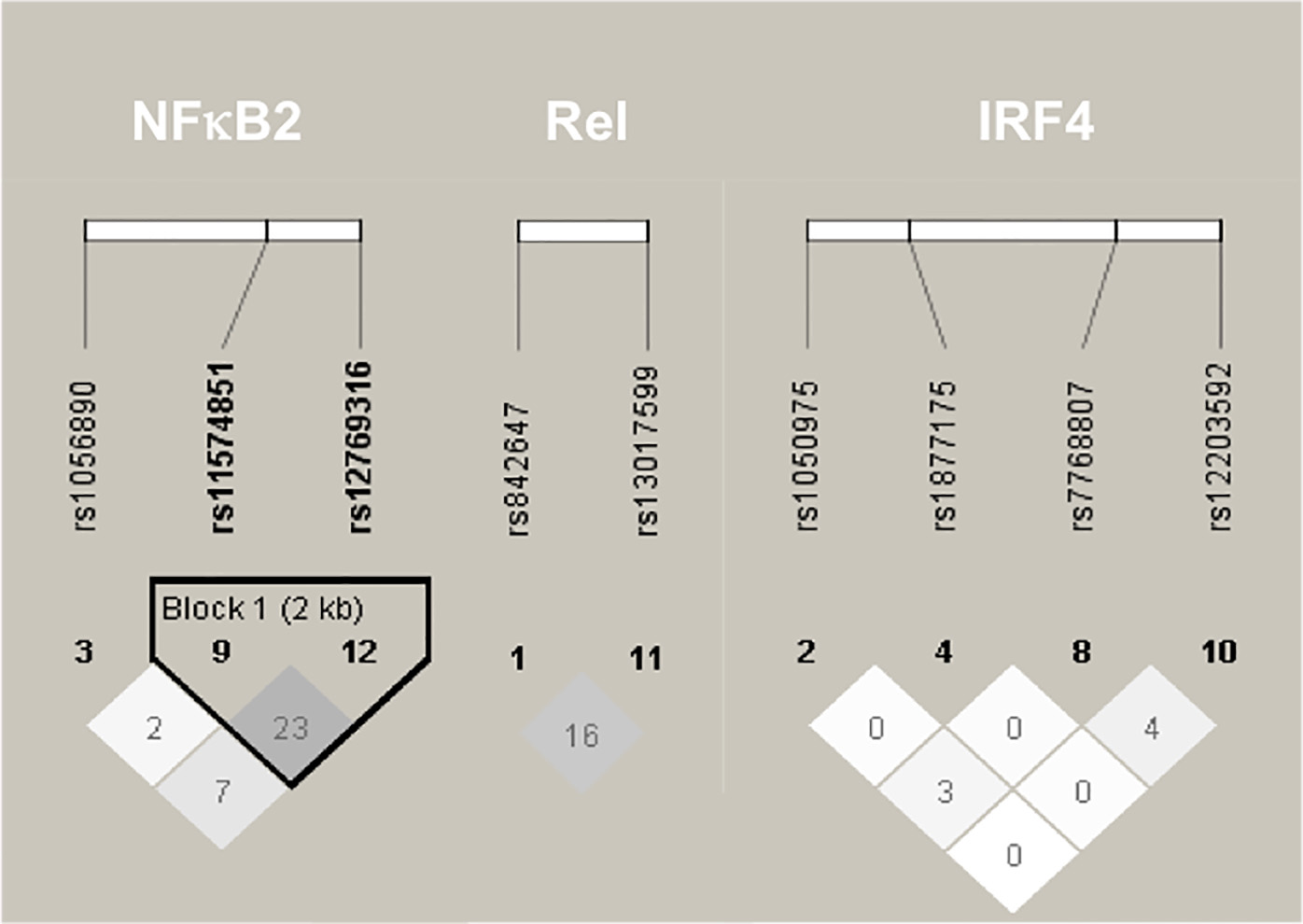

Supplement: Supplementary Figure 1 — Linkage disequilibrium (LD) blocks in the NFκB-related genes calculated in our population. Numbers into squares indicate r2 values. [file Image1.jpg]
